# The Roots of Defense: Plant Resistance and Tolerance to Belowground Herbivory

**DOI:** 10.1371/journal.pone.0018463

**Published:** 2011-04-06

**Authors:** Sean M. Watts, Craig D. Dodson, O. J. Reichman

**Affiliations:** 1 Environmental Studies Institute, Santa Clara University, Santa Clara, California, United States of America; 2 Department of Physical and Environmental Sciences, Mesa State College, Grand Junction, Colorado, United States of America; 3 Department of Ecology, Evolution and Marine Biology, University of California Santa Barbara, Santa Barbara, California, United States of America; Duke University, United States of America

## Abstract

**Background:**

There is conclusive evidence that there are fitness costs of plant defense and that herbivores can drive selection for defense. However, most work has focused on above-ground interactions, even though belowground herbivory may have greater impacts on individual plants than above-ground herbivory. Given the role of belowground plant structures in resource acquisition and storage, research on belowground herbivores has much to contribute to theories on the evolution of plant defense. Pocket gophers (Geomyidae) provide an excellent opportunity to study root herbivory. These subterranean rodents spend their entire lives belowground and specialize on consuming belowground plant parts.

**Methodology and Principal Findings:**

We compared the root defenses of native forbs from mainland populations (with a history of gopher herbivory) to island populations (free from gophers for up to 500,000 years). Defense includes both resistance against herbivores and tolerance of herbivore damage. We used three approaches to compare these traits in island and mainland populations of two native California forbs: 1) *Eschscholzia californica* populations were assayed to compare alkaloid deterrents, 2) captive gophers were used to test the palatability of *E. californica* roots and 3) simulated root herbivory assessed tolerance to root damage in *Deinandra fasciculata* and *E. californica*. Mainland forms of *E. californica* contained 2.5 times greater concentration of alkaloids and were less palatable to gophers than island forms. Mainland forms of *D. fasciculata* and, to a lesser extent, *E. californica* were also more tolerant of root damage than island conspecifics. Interestingly, *un*damaged island individuals of *D. fasciculata* produced significantly more fruit than either damaged or undamaged mainland individuals.

**Conclusions and Significance:**

These results suggest that mainland plants are effective at deterring and tolerating pocket gopher herbivory. Results also suggest that both forms of defense are costly to fitness and thus reduced in the absence of the putative target herbivore.

## Introduction

Most theories on the evolution of plant defense are based on the premise that the competing demands of growth, reproduction, and defense constrain patterns of energy allocation (e.g. Carbon/Nutrient Balance [1], Resource Availability Hypothesis [2], Growth/Differentiation Balance [3]). Accordingly, research in this area over the past two decades has established that herbivores can drive selection for defense and that there are fitness costs associated with defense [4], [5], [6], [7], [8]. Any trait that confers a fitness benefit to a plant in the presence of herbivores can be considered a defense [9], but traditionally, defense referred specifically to resistance traits to deter herbivores (e.g. antibiosis or non-preference strategies [10], [11]). From this perspective, tolerance traits to minimize the impact of herbivory after it has occurred (e.g. compensatory growth or reproduction *sensu* [12], [13]) were considered alternative strategies that correlate negatively with resistance [14]. The logic behind this tradeoff was that selection for tolerance would be minimal in resistant plants, whereas if resistance traits were more costly than regrowth, then tolerance would be favored [13]. There is some evidence that this tradeoff occurs, but increasing evidence suggests the maintenance of a mix of resistance and tolerance traits is common [15]. This suggests that plant defense is better viewed as multifaceted, with defense syndromes composed of suites of covarying traits including: low nutritional quality, toxins, escape through phenology, regrowth capacity, and the recruitment of natural enemies [15], [16], [17]. Therefore, tradeoffs should operate on the evolution of plant defense at two levels: 1) between growth/reproduction and the net energetic costs of a plant's defense syndrome and 2) *among* the traits that comprise the defense syndrome [15].

Given the role of belowground plant structures in resource acquisition, metabolite synthesis and storage, impacts by root herbivores should be especially relevant to our understanding of tradeoffs within defense syndromes and between defense and growth/reproduction [13], [18]. However, studies on belowground herbivory have been limited, in part, by the difficulties of conducting experiments in subterranean systems and of excluding belowground herbivores [19]. In this study we take advantage of an island-mainland study system to compare defense in populations of two plant species with and without a history of exposure to root herbivores.

Of the relatively few studies on belowground herbivory most focus on insect herbivores and the impacts of vertebrate root herbivores are often overlooked as too generalized to have much influence on the evolution of plant defense [19]. In addition, studies of natural and simulated vertebrate root herbivory demonstrate limited tolerance to root damage due to its severity [20], [21], [22], [23]. For example, Reichman & Smith [24] have shown that up to 75% removal of total aboveground plant material has less impact on biomass and flower production than just 25% root loss in a biennial (*Tragopogon dubius*, Asteraceae). However, pocket gophers (Geomyidae) and their ecological cognates on other continents have a major influence on individual plants and plant communities through direct consumption and indirectly through habitat modification [25]. These subterranean rodents are very abundant in western North America, spend most of their lives belowground and specialize on consuming roots [26], [27], [28], [29]. As such, studies of pocket gophers offer a window on the responses of plants to this widespread form of root herbivory.

Most studies on gopher herbivory have focused on plant tolerance or plant community responses to the activities of belowground herbivores. To our knowledge, no work has been conducted to investigate plant deterrence of pocket gophers. This study provides an initial assessment of the influence of pocket gophers (*Thomomys bottae*, Geomyidae) on defense in a subset of species likely to experience the direct effects of pocket gopher herbivory in California grassland communities. Pocket gophers are widely distributed and often reach high densities in California grasslands [27], [28]. Moreover, specimens of *T. bottae* are the most frequently uncovered remains in the tarpits of Rancho La Brea in Los Angeles County and their fossorially-adapted morphology appears essentially unchanged for 4.6 my ([30]). Andersen & MacMahon [31] found that gophers may consume more than 30% of total belowground annual primary productivity in Utah meadows, where they occurred at densities lower than the mean density observed in California grasslands [32]. Considering the current and historic abundance of these belowground herbivores, plants in mainland California would be expected to have evolved defenses against gopher herbivory.

In contrast to the California mainland, there is no current or fossil evidence of pocket gophers (*Thomomys bottae* Geomyidae) [33], although the Channel Islands were inhabited by the dwarf mammoth, *Mammuthus exilis* for nearly 50,000 of the past 60,000 years and livestock were introduced ∼150 years ago [34], [35], [36]. The northern four Channel Islands existed as one land mass (“Santarosae”) during the Wisconsin glacial period (0.06–0.01mya) and parts of the largest two islands (Santa Cruz and Santa Rosa) have been above sea level for the past 0.5my. Although Santarosae may have been separated from the mainland by as little as 8 km during sea-level minimum, there is no geologic evidence that they have ever been connected to the mainland [37], [38]. Thus, the Channel Islands provide a rare opportunity to assess the defense traits of plants that have evolved in the absence of gopher herbivory. Mainland plant populations with high densities of pocket gophers would likely benefit from the ability to either deter or tolerate root herbivory, whereas adaptations to gopher herbivory would presumably be less important in island populations that have evolved in the absence of gophers. Plant tolerance has not been examined in this system, but Bowen & van Vuren [39] showed that Channel Island forms of six chaparral shrubs had significant reductions in aboveground deterrent tannins and were more palatable to sheep than similar mainland species.

The peculiarities of islands have always fascinated naturalists [40], [41], [42], [43], [44]. Although it is difficult to avoid ‘pseudoreplication’ [45] when using islands in comparative studies, islands and other ‘natural experiments’ are often the only realistic means of investigating some questions or promoting further investigation [46]. Islands are especially important when investigating the long term selective influence of otherwise ubiquitous herbivores or competitors. The relaxation of defensive traits in island plants has been demonstrated by the loss of ant-defense mutualisms in island species of *Cecropia* [47], [48] and more recently in reductions in chemical defenses in island forms of red cedar, *Thuja plicata* [49]. We sought to extend this body of work belowground and encourage more research into the evolution of plant defense to root herbivores.

In our studies we used island and mainland populations of two native plant species to consider the potential for root herbivores to influence two categories of defense: chemical defense (*Eschscholzia californica* Cham., Papaveraceae) and tolerance (*E. californica* and *Deinandra fasciculata* (DC.) Greene, Asteraceae). Both species are abundant tap-rooted grassland forbs that are commonly eaten by pocket gophers. *D. fasciculata* is an annual species and *E. californica* is a short-lived perennial. These species were chosen, in part, to allow us to detect differences between annuals and perennials in their allocation to deterrence and tolerance. More detail is available as supporting information; see Text S1: *Study Species*. We focus mainly on the overall tradeoff between our study species' defense syndromes and growth/reproduction, but in assessing resistance and tolerance separately we also discuss the potential for independent selection on these traits and their relative importance to annual *versus* perennial species.

*Resistance*- Do mainland populations of *E. californica* possess deterrent compounds that make them less palatable to gophers? Conversely, do island plants, in the absence of gophers, produce fewer deterrent compounds than mainland conspecifics? Conspecifics from one population each on Santa Cruz and Santa Rosa Islands and the adjacent mainland were assayed to compare alkaloid defenses. Captive gophers were used to compare the palatability of plants from two mainland populations and two Santa Cruz Island populations. We predicted that the roots of island plants would contain lower concentrations and fewer individual alkaloid-class compounds and would be more palatable to gophers than the roots of mainland conspecifics. Resistance is usually defined from the herbivore's perspective (i.e. reductions in the fitness of the herbivore); we use the inclusive term resistance to refer to the entire suite of traits directed at deterring herbivores and to distinguish these chemical defenses from tolerance traits that involve compensation after herbivore damage.

*Tolerance*- Are mainland populations more tolerant of root damage than island conspecifics, which have not been exposed to pocket gophers? Simulated root herbivory was applied in two Santa Cruz Island and two mainland populations of both *D. fasciculata* and *E. californica* to compare tolerance to root damage. We predicted that island plants would exhibit greater mortality and lower fecundity in response to root damage than their mainland counterparts.

Root herbivory is especially valuable in studies of tolerance and compensatory regrowth, because it does not directly influence apical dominance (i.e. release of dormant buds from the hormonal suppression of lead meristems), which is an important response mechanism to aboveground grazing.

## Materials and Methods

### Ethics Statement

Research at the University of California Natural Reserve System Coal Oil Point, Santa Cruz Island Reserves was conducted under research application index numbers 768 & 769. Direct permissions were obtained for research conducted at the following sites: Vandenberg Air Force Base, Refugio State Park, Santa Monica Mountains Natural Reserve Area (Charmlee Park- City of Malibu, Topanga Canyon State Park, Leo Carrillo State Park, Pt. Mugu State Park) and Channel Islands National Park (Santa Rosa Island). Pocket gophers were captured and held in the Central Vivarium at UCSB under California Department of Fish and Game research permits #803009-03 & SC-004300. Diet choice experiments were run under UCSB Institutional Animal Care and Use Committee Protocol Authorization #2-00-574.

### Study System

Four mainland sites (M) were used, Vandenberg Air Force Base: N 34°34.0′ W 120°37.8′, Gaviota State Park: N 34°28.4′ W 120°12.9′, Refugio Ranch: N 34°29.6′ W 120°04.1′, Coal Oil Point Reserve: N 34°25.0′ W 119°52.8′. Island sites included one on Santa Rosa Island (SR), Southeast Anchorage: N 33°59.0′ W 120°00.9′ and three on Santa Cruz Island (SC), Christy Airstrip: N 34°01.2′ W 119°50.8′, Campo Raton: N 34°01.1′ W 119°49.0′, and the University of California Field Station: N 33°59.9′ W 119°43.8′.

The California Channel Islands and the adjacent mainland share a mediterranean climate: warm, dry summers and mild, wet, nearly frost-free winters [50]. Sites were paired between the Channel Islands and the adjacent coastal mainland of Santa Barbara County to reflect a range of comparable soil and climatic conditions. During the growing seasons of the experiments (Fall 2000–Summer 2003), Channel Island sites had mean annual temperatures from 13.3–15.6°C and total precipitation from 14.9–54.9 cm/yr. The range of temperatures for mainland sites was 14.5–16.6°C, with total precipitation of 22.6–62.1 cm/yr. Island and mainland soils ranged from clay loam to sandy loam. NH_4_ was below detectable levels at all sites; mainland sites had both the lowest and highest NO_3_ (Gaviota, 4ppm; Vandenberg, 11ppm); all other sites had 5–7ppm NO_3_ (see Text S1: *Study Sites*; see also Table S1 and S2 for mean annual temperatures, precipitation and soil data).

Two species of native California grassland forbs were chosen to represent plants that experience the direct effects of gopher burrowing and root consumption. We studied tap-rooted forbs, as it has been shown that gophers generally prefer these over fibrous rooted grasses [51], [52], [53]. We also chose forbs whose roots commonly grow to the depth of gopher feeding tunnels (∼10–20 cm [54], [55]). Using these criteria two species were chosen for the study: one annual, common tarweed, (*Deinandra fasciculata*; formerly *Hemizonia fasciculata*) and one short-lived perennial, the coastal variety of the California poppy (*Eschscholzia californica*) (see Text S1: *Study Species*).

### Resistance

Resistance in island and mainland conspecifics of *E. californica* was assessed through: 1) chemical assays of alkaloid content (alkaloids are well described herbivore deterrents [3], [11]) and 2) diet choice experiments with captive gophers.

#### Alkaloid analysis

Percent by mass of basic alkaloids and the number of individual basic alkaloid-class compounds were separately assayed for roots and shoots. Five individuals of *E. californica* were collected between 28 April and 7 May 2002 from each of the following sites: SE Anchorage (SR), C. Raton (SC), and C.O. Point (M). Individuals chosen were non-flowering plants exhibiting little or no aboveground herbivory and no gopher herbivory. *E. californica* synthesizes a wide variety of alkaloid chemicals in all plant parts although concentrations tend to be higher in roots [56]. Roots and shoots were separated in the field to prevent possible transfer of materials between them. Because the basic alkaloids isolated in this process are quite stable, samples were shade-dried separately for ∼1month in paper bags and analyzed over the summer of 2002 at Mesa State College. Entire samples of either roots or shoots were milled to a fine powder and crude mixtures of basic alkaloids were isolated by differential pH extraction. The masses of these crude mixtures were measured and the percentage of basic alkaloids by mass was calculated based upon dry weight of plant material. Two methods were used to determine the number of individual basic alkaloid-class compounds in the mixture: a 300 MHz proton NMR spectrum was collected for each crude base sample (JEOL Eclipse 300) and a portion was used for TLC analysis on silica gel and visualized using short wave UV absorbance, long wave UV fluorescence and an iodoplatinic acid alkaloid specific spray reagent (for details on extraction, NMR, and TLC, see Text S1: *Alkaloid Analysis*).

#### Diet Choice Experiments

Ten captive pocket gophers were used to compare the palatability of *E. californica* from the island and mainland sites (after [57]). Between 29 April and 10 May 2003, five gophers each were captured at El Capitan Ranch (N34°28.046′ W119°59.275′) and the Del Sol Vernal Pool Reserve (N34°24.530′ W119°52.682′) in Santa Barbara County. The seven males and three females weighed between 81.4 g to 211.4 g. Animals were housed in separate polycarbonate rat tubs (48.3×26.7×20.3 cm; #R20PC; Ancare, P.O. Box 814, Bellmore, NY 11710) at the UCSB vivarium (70–72°F, 12 hr light cycle; Animal Resource Center). During an equilibration period (from capture to 19 May 2003) gophers were provided with 2 pellets of laboratory food per day (Purina Rodent Chow no. 5001) and as much root and shoot material of store-bought vegetables as they could eat.

On 16 May 2003 between 1:00 and 8:00pm the roots of ∼35 undamaged individuals of *E. californica* (∼30 cm tall) were collected at two Santa Cruz Island sites (C. Raton, Field Station) and two mainland sites (Vandenberg, C.O. Point). Roots were immediately stored in plastic bags on ice for 48 hrs before being stored in a cold room (48–52°F). All feeding trials were performed between 19 and 30 May 2003. The palatability of roots from island and mainland populations was assessed with nine 4 hr diet choice trials (which included comparisons of climatically similar and divergent populations; see Text S1: *Study Sites* and *Diet Choice Experiment*). For each trial, 6–8 roots were used from island or mainland sources. To distinguish between these two sets of conspecific roots, roots from each source were scored longitudinally with a knife either once or twice (on opposite sides) to a depth of 1–2 mm (scoring was assigned randomly for each trial). Each root was cut into ∼3 g pieces and distributed to food bowls. To avoid any visual bias in root selection, the island and mainland root pieces provided to each gopher were similar in length and diameter. After recording the initial weight of roots provided, gophers were allowed to feed for four hours, at which time remaining food was recovered. Cached roots (hidden in bedding and nest boxes) and declined roots (left in food bowls) were weighed separately. This total (cached + declined) was subtracted from the amount initially given to determine the amount consumed. Sample pieces of island and mainland roots left on *Care*FRESH® bedding indicated that weight loss due to evaporation was minor relative to gopher preferences and were similar across all populations.

### Tolerance

In spring 2003, simulated root herbivory experiments were conducted on island and mainland populations. Two island and two mainland populations were used for each species: *E. californica*- C. Raton, Field Station, Vandenberg, and C.O. Point; *D. fasciculata*- Christy, Field Station, Gaviota, and Refugio. At each population at least 24 pairs of non-flowering, undamaged plants were marked; there were no significant differences in the initial size of control and root-damaged plants at any site (one-way ANOVA on ln (initial plant volumes); *D. fasciculata*: *F* = 0.2749, *P* = 0.60, *E. californica*: *F* = 0.1746, *P* = 0.68). In anticipation of gopher activity in mainland populations, 30 pairs (instead of 24, as in island populations) were chosen and any pairs experiencing gopher damage (mounding or tunneling) were excluded from analysis. A root damage treatment simulating gopher herbivory was applied to one individual of each pair with a 7.5 cm diameter bore Dutch Mud Soil Auger. A series of calibration treatments for each species was used to determine the aboveground size of plants with roots that reached depths of at least 15 cm. The tip of the auger was placed approximately 20 cm from the stem and pointed towards the base of the plant at an approximately 35° angle from horizontal. The auger was then driven into the soil for at least 25 cm, which placed its tip at 11–14 cm directly below the stem base, a depth similar to gopher foraging burrows (see Text S1: *Tolerance Experiment Design*; see also Fig. S1 and S2 for treatment demonstration). Prior to the simulated herbivory, an initial census (census 0) was taken to establish baseline data for each individual. Two post-treatment censuses (censuses 1 and 2) were performed approximately 45 and 90 days after census 0 to assess the growth, survivorship and reproduction of control and root-damaged plants. Reproduction was measured as total number of seeded inflorescences (*D. fasciculata*) or pods (*E. californica*). As a member of the Asteraceae, *D. fasciculata* has composite flowers with five ray and six disc florets per inflorescence. These fruiting heads consistently had five ray achenes and 3–6 smaller pappose disc achenes. *Eschscholzia californica* has simple flowers; fruits are cylindrical pods, 3–7 cm long, with 20–40 seeds per pod.

A common garden study was initiated in 2001 in a mainland old-field at a private residence in Montecito, California, USA (Rivenrock: N 34°26′ W 119°38′). *D. fasciculata* seed was collected from three mainland and three Santa Cruz Island sites in summer 2000 and homogenized into island and mainland packets. The common garden was planted in late January 2001 within a gopher exclosure (hardware cloth sunk ∼1 m belowground). All measurements followed the protocol for the tolerance experiments, although there were no damage treatments. Recruitment was too low to conduct the tolerance experiments, however, growth and reproduction of island and mainland plants were recorded to evaluate genotypic vs. phenotypic population responses under common conditions.

### Statistical Analyses

All statistics, except Tukey HSD tests [58] for alkaloid data, were performed using SPSS software (SAS Institute, Cary, North Carolina, v 5.1). Diet choice preferences (amount eaten, cached, and declined) were analyzed using repeated measures ANOVAs across all trials. Survivorship was analyzed using a parametric survival fit to plant lifespan according to census dates (censored for individuals alive at the final census). Survivorship data were fit to a Weibull distribution, but lognormal and exponential distributions provided the same results. For reproduction, treatment mortality in island populations resulted in unbalanced samples if dead individuals were ignored and skewed data if dead individuals were included (i.e. right-skewed due to zeros). Mixed-model ANOVAs on final census fecundity were, therefore, performed on ranked data with and without zero data (other nonparametric analyses produced qualitatively identical results). The basic mixed model included source (island or mainland), treatment (control or root-damaged), their interaction as fixed effects, and population nested within source as a random effect. A Bonferroni correction for comparing island and mainland data required significance at α = 0.025.

## Results

### Resistance: Alkaloid analysis

**Figure 1:**
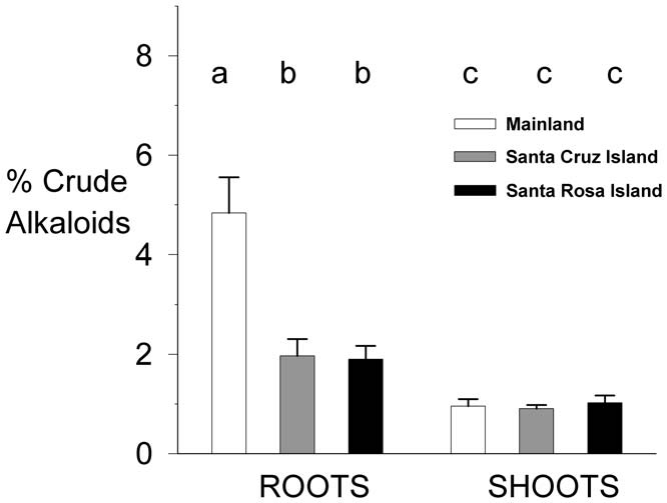
Summary of plant chemical analysis for mainland versus island root material of *Eschscholzia californica*. Values plotted are mean percent by mass of crude alkaloids of five plants per site (±1 SE). Letters indicate significant differences between bars within root or shoot categories.

As predicted, roots from the mainland population (C.O. Point) of *Eschscholzia californica* had significantly higher alkaloid content (mean: 4.84%) than mainland shoots (mean: 0.96%) or all plant parts from island populations (grand mean: 1.45%). Although chemical assays included only one mainland site, samples from two separate islands had very similar root and shoot alkaloid content. The alkaloid content of roots from the Santa Rosa (SE Anchorage, 1.90%) and Santa Cruz (C. Raton, 1.97%) island populations were significantly lower than C.O. Point roots (*q*_0.05,12,3_ = 3.77; C.O. Point vs. SE Anchorage *q* = 6.05; vs. C. Raton *q* = 5.92) and not significantly different from each other (*q* = 0.14). The percent alkaloids by mass in the roots were also 2–3 times more variable by range for C.O. Point plants than for island plants. Basic alkaloid content for shoots did not significantly differ among sites (*q*_0.05,11,3_ = 3.82; SE Anchorage vs. C. Raton *q* = 1.01; vs. C.O. Point *q* = 0.57; C.O. Point vs. C. Raton *q* = 0.39; see Fig. 1). Benzophenanthrines, which are water soluble at all pH, were also found in shoots; however, they were present only in small amounts in roots. The consistency of results from NMR and TLC indicate that the only major components of our extracts were alkaloids (see Text S1: *Alkaloid Analysis*).

TLC and proton NMR data also indicated that island root samples all contained the same two alkaloids in similar proportions with a small amount of a third compound in one sample. In contrast, C.O. Point (mainland) root samples were more complex with at least five different alkaloids present in varying proportions and differing numbers of compounds.

### Resistance: Diet Choice Experiment

**Figure 2:**
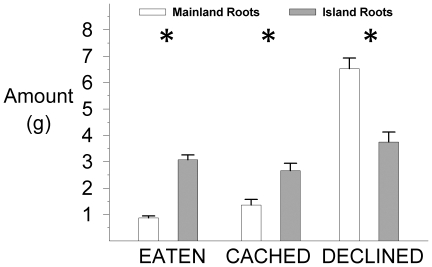
Summary of preferences of gophers for mainland versus island root material of *Eschscholzia californica*. Values plotted are pooled means of amounts of root material eaten, cached or declined by 10 gophers for 9 trials (±1 SE). * indicates significant difference between preferences for island and mainland root material.

In addition to the mainland and Santa Cruz Island populations used in the alkaloid assays, an additional mainland population of *E. californica* was used for the diet choice study to mirror climatic differences between island sites and support the alkaloid analyses. Pocket gophers showed a strong preference for the root material of both island populations of *E. californica* over roots from either mainland population. The amounts of material cached and declined are not independent of the amount eaten (because all sum to the amount offered), however, they are presented separately as each corresponds to a discrete foraging decision (Fig. 2). Repeated measures ANOVAs across all 9 trials demonstrate that gophers: 1) ate more island root material than mainland material (*F*_1,16_ = 19.3, P<0.001), 2) cached more island root material than mainland material (*F*_1,16_ = 6.6, P = 0.021), and 3) declined more mainland root material than island material (*F*_1,16_ = 13.6, P = 0.002). All individual trials indicated the same preference for island material, regardless of the comparison of climatically similar or divergent populations.

### Tolerance: *Deinandra fasciculate*

Across all censuses, there was a greater reduction in island root-damaged plant survivorship (relative to controls) than in mainland populations, where there were no significant differences between control and root-damaged plant survivorship (parametric survival fit: *X* ^2^ = 9.1, *P* = 0.003; see Table S3 for survivorship data).

**Figure 3:**
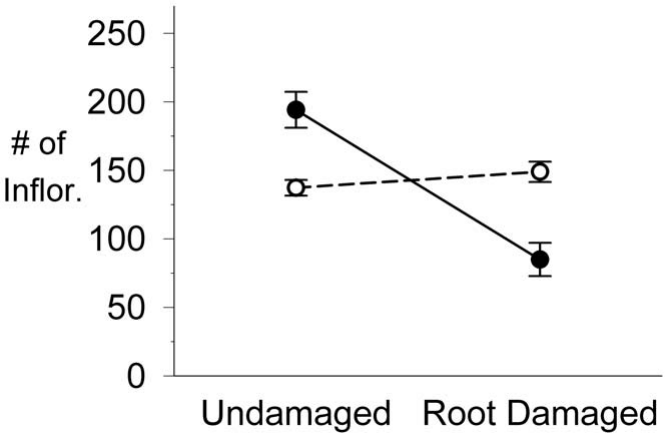
Mean reproduction in control and root-damaged plants of mainland and island *Deinandra fasciculata*. Values plotted are means of total number of seeded inflorescences per plant in the final census (±1 SE). Data shown exclude plants that failed to reproduce; including these individuals would not change values for control plants and increases disparity between root-damaged plants. Mainland sites (n = 2) indicated with open circles, island sites (n = 2) with closed circles.

As mentioned in the Materials and Methods (*Statistical Analyses*), disproportionate mortality in island treatment plants resulted in unbalanced or skewed samples for fecundity (i.e. dead plants produce no seed), however, all methods of analysis yielded the same results: highly significant interactions between source (island vs. mainland) and treatment (damaged vs. not; mixed ANOVA on ranked data: *F*_1,199.9_ = 87.73, *P*<0.001). There was no significant difference in reproduction between mainland control and treatment plants, whereas island treatment plants had significantly lower reproduction than controls (Fig. 3). Interestingly, island control plants produce more flowers and fruits than mainland controls, but suffered more from root damage.

The overall pattern of greater reproduction in island *versus* mainland plants was supported by the 2001 common garden study (see Table S4; Census 3, *U*_0.05(1)3,5_ = 14; Census 4: *U*_0.10(1)3,5_ = 13). Thus, island plants displayed the ‘overproduction’ phenomenon noted in the *in situ* tolerance experiments despite being grown on the mainland. In fact, the common garden study showed a greater disparity between island and mainland control plants than in the field (common garden: Island ∼2.7 times more fecund than mainland vs. *in situ*: Island 1.4 times more fecund than mainland).

### Tolerance: *Eschscholzia californica*

The survivorship results for *E. californica* were similar to *D. fasciculata*: across all censuses, a greater reduction in island root-damaged plant survivorship (relative to controls) than in mainland populations, where there were no significant differences between control and root-damaged plants (parametric survival fit: *X* ^2^ = 6.1, *P* = 0.013; see Table S3).

**Figure 4:**
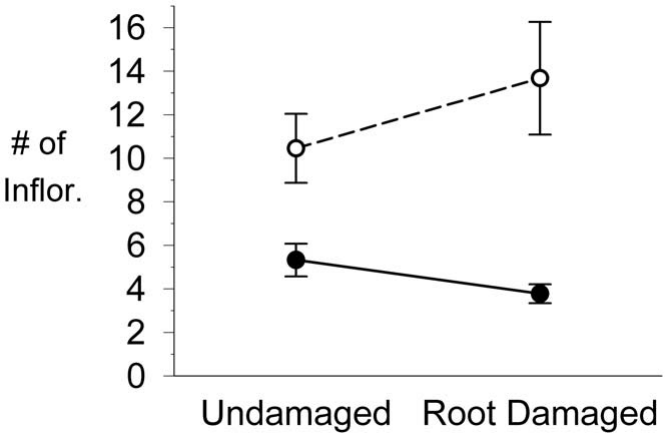
Mean reproduction in control and root-damaged plants of mainland and island *Eschscholzia californica*. Values plotted are means of total number of seeded inflorescences per plant in the final census (±1 SE). Data shown exclude plants that failed to reproduce; including these individuals similarly reduced all means yielding the same results. Mainland sites (n = 2) indicated with open circles, island sites (n = 2) with closed circles.

Although there was a trend toward greater reductions in root-damaged plant fecundity in island populations than on the mainland (mixed ANOVA on ranked data: *F*_1,192_ = 3.8, *P* = 0.053; see Fig. 4), it was not significant with a Bonferroni correction (α = 0.025).

## Discussion

There were several challenges to testing the hypotheses that drove this research. The first is that the occurrence of gophers is, of course, not the only difference between the islands and the mainland. The locations also vary somewhat in climate and soils, however, the consistency of results from separate species, populations, experiments and in the common garden study suggest a major role for the historic presence/absence of pocket gophers. The ideal design would have also included reciprocal transplants, but mainland genotypes could not be introduced to the islands and transportation to and on the islands was limiting. To try to address these limitations, we used three different approaches to test the hypotheses, and, in all but the alkaloid analyses, we used two replicate populations each from Santa Cruz Island and the mainland. Finally, although running the experiments in the mainland common garden was precluded by space limitations and low recruitment of sown seed, *D. fasciculata* plants that did grow in the common garden showed the same pattern of greater productivity in island *versus* mainland control plants witnessed in the tolerance experiment.

We cannot definitively ascribe these results to genetic differences between island and mainland populations, but given the costliness of root damage and the extent of gopher herbivory on the mainland, the results from this study are a strong indication that pocket gophers have a selective influence on root defense. Below, we discuss in greater depth the findings from our alkaloid assays, diet choice studies, and root damage experiments.

### Resistance

It is notable that *E. californica* roots from both Santa Rosa and Santa Cruz Island showed similar reductions in not only the mass, but the variety of basic alkaloids relative to the mainland population (see Fig. 1). We lacked authentic standards and budget to identify the specific alkaloids present in our extractions and alkaloid diversity could correlate with concentration, however, with a greater variety of alkaloid structures, mainland plants would have a greater chance of producing compounds that are chemically active against a given consumer [59]. It is also notable that shoot levels of basic alkaloids were similar in all sites, suggesting that high root alkaloid production in mainland plants targets root herbivores.

Given the small sample size of our chemical assays, these interpretations should be read with caution; however, our diet choice experiments included an additional mainland population and provide further support for the hypothesis that defense chemicals in mainland forms are effective against the putative target herbivore. Gophers preferentially ate and cached island roots, while declining (and in several instances urinating on) mainland samples. Although it is possible that the diet choices reflected a preference for higher quality foods (we did not assess energy or protein content in the roots), research on other vertebrate herbivores has shown greater focus on avoiding unsuitable foods than consuming the highest quality foods [60].

### Tolerance

The gopher-plant interaction is an especially appropriate system for studying tolerance because root herbivory directly affects the organs involved in resource acquisition and storage that would normally be enlisted in tolerance. Calibration treatments (see Materials and Methods) indicated that our damage treatments removed approximately 25% of the root volume. Damaged individuals in both island and mainland populations showed the same initial wilting response to the simulated root damage treatment, with mainland plants recovering significantly more often and more completely than island plants. For *E. californica*, the basal rosette of leaves began to wilt within an hour of treatment, and growth in damaged individuals surviving to the next census was always from new meristems- typical of an herbaceous perennial resuming growth after dormancy. In the case of *D. fasciculata*, the damaged plants wilted, with their main stem bending towards the ground. With both island and mainland survivors, however, recovery resulted in a distinctive ‘S’-shaped kink in the main stem. Despite these obvious signs of severe root damage in island and mainland treatment plants, *mainland* survivors of both species displayed complete compensation after root damage.

It is interesting that island populations of *E. californica* displayed high tolerance to root damage. Energy storage in this perennial may have limited our ability to detect reduced tolerance and by testing *E. californica* over a single season we would have missed any impacts that carried over to subsequent seasons- as has been shown in both theoretical and empirical studies of perennial plants [61], [62]. However, these results also support the suggestion that perennial plants retain some compensatory ability as a byproduct of iteroparity and the near certainty of either herbivore or environmentally induced damage over the course of their lifespan [1], [63], [64], [65], [66].

In contrast to *E. californica*, *D. fasciculata* is an annual plant with a semelparous, ‘Big Bang’ reproductive strategy. The high mortality observed in island root-damaged plants and the severely reduced reproduction of surviving individuals suggest that island populations are quite intolerant of root damage. In addition, mainland populations showed exact (or slight over-) compensation. However, this ability to compensate seems to come at a cost for mainland plants. On average, island control plants produced 1.4 times as many seeded inflorescences as mainland control plants. On the other hand, greater productivity in island plants appears to make them quite vulnerable to root damage, as mean productivity of surviving root-damaged plants was 2.3 times lower than that of undamaged controls (see Fig. 3). The unusually high productivity in island control plants suggests a tradeoff where resources that might formerly have been dedicated to defense are released for greater growth and reproduction. In the absence of gophers on the island, individuals with reduced deterrence and tolerance would have a selective advantage over those retaining defenses against gophers. The potential role of environmental differences between the mainland and island sites appears to be minimal: 1) the greater productivity of island plants was even more dramatic in the mainland common garden and 2) if island populations experienced better growing conditions, then root-damaged plants on islands would be expected to benefit from these conditions as well, but this was not the case. Clearly we need more research on the specific mechanisms of compensation (e.g. compensatory root regrowth vs. efficient resource storage and reallocation); however, given the severity of this root damage, it is remarkable that mainland *D. fasciculata* plants are able to compensate at all for such damage.

### Conclusions

Several theories of optimal defense state that inherently fast-growing plants in relatively high resource environments have high opportunity costs for investments in defense due to the premium placed on fast, competitive growth [1], [2], [10], [11]. Both of our study species are relatively fast-growing and our research appears to demonstrate this overarching tradeoff between defense and growth, but it is also interesting that resistance and tolerance traits in our fast-growing annual and short-lived perennial seem to have responded independently to release from root herbivores. In the past, resistance and tolerance tended to be viewed as mutually exclusive adaptive strategies with the shared goal of minimizing the negative impacts of herbivory [3], [67], [68]. However, studies that simultaneously consider resistance and tolerance in plants to herbivores provide evidence for the stable maintenance of both at either the population or individual level [15], [17], [69], [70], [71]. It is unfortunate that we did not include *D. fasciculata* roots in our resistance studies, but island populations of this annual displayed reduced tolerance and evidence for dramatically increased growth. In contrast, we found reduced chemical defenses and increased palatability in island forms of the perennial *E. californica*, but the apparent retention of tolerance, which would be consistent with the general importance of tolerance to longer-lived plants.

Given the inherent limitations of an island-mainland design, these conclusions are tentative, but we hope that they will encourage more investigators to overcome the obstacles to studying belowground plant-herbivore interactions. Considering the costliness of root damage to plant resource acquisition and storage, it is important that we continue to compliment our knowledge of aboveground plant defense with increased understanding of belowground herbivores and the trade-offs involved in root defense.

## Supporting Information

Text S1 **Detailed Methods.** Supplementary information on study species, study sites, alkaloid analysis, diet choice experiment and tolerance experiment design.
(DOC)

Figure S1 **Demonstration of the tolerance treatment.** **A** “Dutch Auger” used in the experiment. **B** after root damage treatment applied. The plant shown receiving the treatment is telegraph weed (*Heterotheca grandiflora*, Asteraceae).
(TIF)

Figure S2 **Comparison of actual and simulated root herbivory.** **A** Plugged laterals of an actual gopher burrow (to the right and below plant). **B** Plugged hole resulting from the treatment depicted in Figure S1 (to the right of plant). The wilting of the plant in Figure S2A is a typical reaction to gopher damage that would also occur within an hour of the treatment in Figure S2B.
(TIF)

Table S1 **Mean annual temperature (°C) and total annual precipitation (cm) at representative mainland and island sites.**
(DOC)

Table S2 **Soil data for each mainland and island site (SE Anchorage is on Santa Rosa Island).**
(DOC)

Table S3 **Survivorship of** ***Deinandra fasciculata*** **and** ***Eschscholzia californica*** **in tolerance experiment by site (Note: table does not include mainland pairs dropped from analyses due to gopher mortality).**
(DOC)

Table S4 **2001 Common Garden Results: Mann-Whitney tests on ranked total number of flowers and fruits (TFF) in undamaged island (I1–I5) and mainland (M1–M3)** ***Deinandra fasciculata*** **in 2001 (censuses 3 & 4).**
(DOC)
