# Supplementary material for: The Roots of Defense: Plant Resistance and Tolerance to Belowground Herbivory
Source: PLoS One. 2011 Apr 6;6(4):e18463. doi: 10.1371/journal.pone.0018463 (PMC3071833; doi:10.1371/journal.pone.0018463)
Supplement: Text S1 — Detailed Methods. Supplementary information on study species, study sites, alkaloid analysis, diet choice experiment and tolerance experiment design. (DOC) [file pone.0018463.s001.doc]

**Text S1.** **Detailed Methods.** Supplementary information on study species, study sites, alkaloid analysis, diet choice experiment and tolerance experiment design.

*Study Species*

The list of potential candidates for this study was determined by the following criteria: 1) all candidates are California natives, 2) all are common in grasslands on at least two of the northern four Channel Islands as well as coastal mainland Santa Barbara County, 3) none were rare or threatened species, and 4) all commonly grow to depths that place them in the feeding zone of pocket gophers. The depth of gopher feeding tunnels (~10 – 20cm) represents a compromise between soil depths that maintain the structural integrity of the tunnel and access to root biomass, which decreases with soil depth [1,2,3]. With the exception of gopher damage on the mainland, herbivory on these species was similar and low between island and mainland sites. These herbivores included spittle bugs (*Aphrophora* spp., Cercopidae) and grasshopper species (*Dissosteira* spp., Acrididae) [4]. Mainland sites were also inhabited by mule deer (*Odocoileus hemionus*; especially Vandenburg), Santa Cruz Island has feral pigs (*Sus scrofa*), and Santa Rosa Island has introduced mule deer and elk (*Cervus elaphus*). Neither *D. fasciculata* nor *E. californica* experienced any herbivory by feral pigs (though occasional trampling occurred), but elk and deer occasionally removed shoots of *E. californica* at both Vandenburg and Santa Rosa Island, SE Anchorage. No plants used in the diet choice and chemistry experiments were damaged and tolerance plants were only rarely damaged (i.e. minor leaf clipping by insects, less than ¼ of an individual leaf).

The variety of ***Eschscholzia californica*** (Papaveraceae) used in the study is a short-lived perennial forb that forms a deep taproot (2 – 4cm in diameter at the soil surface, often >30cm in depth). This coastal variety has a protracted reproductive season, and while fruit production peaks in spring, some flowering may occur as early as January and continue through the summer. In spring xerochastic fruit pods begin to burst and seed is ballistically dispersed for up to ~1m. Gophers actively feed on *E. californica* (*pers. obs.*).

***Deinandra fasciculata*** (Asteraceae) is a common annual forb in coastal grasslands. After germinating in mid winter it grows for several months as a simple, erect forb, developing a root system with a weakly dominant central taproot (0.5 – 1.5cm in diameter at the soil surface, 20 – 30cm in depth). During this initial vegetative stage *D. fasciculata* allocates water and carbohydrates to root storage ([5]- studying *D. conjugens*, a similar, sympatric congener). The highest risk of gopher herbivory also occurs during this season. *D. fasciculata* is one of three forbs that dominate the spring diet (60 – 70% forbs) of gophers in San Diego County (*T. bottae sanctidiegi*)- the other two species are non-natives (*Erodium botrys* and *Hypochaeris glabra* [6].

With the onset of summer drought the erect herbaceous habit of these plants is lost as they become bushy, woody-stemmed and covered with sticky exudates. The large cauline leaves senesce, their photosynthates are translocated and branches begin to extend from the upper leaf axils to form large cymes of, sometimes, several hundred composite flowers. Pocket gophers in mediterranean-type climates burrow most actively during the wet growing season (winter-spring) and shift to relying on caches and some aboveground forage during the dry, summer months, when the soil becomes difficult to excavate [7,8]. By late April, gophers (indeed most herbivores) avoid *D. fasciculata* as these forbs shift to a reproductive stage with increased chemical and structural defense [5,6]. Thus, the risk of root herbivory declines as *D. fasciculata* begins to shift to reproduction. By late July most individuals have senesced and the achenes are passively released from the receptacles.

*Study Sites*

Mean annual temperature (° Celsius) and total annual precipitation (rainfall in centimeters) are listed for a range of representative island and mainland sites (see Table S1). “Lompoc” represents Vandenberg AFB and Gaviota SP. “Santa Barbara Airport” data represents the mainland sites of Refugio Ranch and C.O. Point Reserve. “Christy Airstrip” data represents the Santa Rosa Island site of SE Anchorage, and the Santa Cruz Island sites of Christy Airstrip and C. Raton. “Field Station” data were collected within 1 mile of the UC Field Station.

Both island and coastal mainland sites represent a gradient from cool, windy, heavily maritime to warmer conditions, with less fog, but more rain. However, because daily fog inundation was not included in the precipitation estimates, the heavily maritime influenced sites (SE Anchorage, Christy Airstrip, Campo Raton, and Vandenberg AFB) are likely to receive more water via fog drip than these data suggest. I was unable to obtain weather data for the Vandenberg site, thus the weather data listed below (“Mainland Lompoc”) are slightly warmer and drier than the actual site conditions. Lompoc is approximately 10 miles inland and to the northwest of the Vandenberg site, which is 200 – 300m from the shore at Point Arguello.

Santa Cruz Island, Christy Airport weather data provided by: John B. Wall, Weather Observer/ Technologies Manager, College of Social & Behavioral Sciences, Department of Geography, California State University, [John.B.Wall@csun.edu](mailto:john.b.wall@csun.edu). Lompoc & Santa Barbara Airport weather data provided by: Joel Michaelson, Professor, Climatology/Meteorology, Department of Geography, Division of Mathematical, Life, and Physical Sciences, University of California, Santa Barbara, [Joel@geog.ucsb.edu](mailto:Joel@geog.ucsb.edu). UC Field Station weather data provided by: Lyndal Laughrin, Director, Santa Cruz Island Reserve, University of California, Santa Barbara, [Laughrin@lifesci.ucsb.edu](mailto:Laughrin@lifesci.ucsb.edu).

Soil composition and NO_3_ concentrations are listed for island and mainland sites (see Table S2); NH_4_ is not reported as it was below detectable levels at all sites. Soil samples (7cm diameter x 5 – 15cm depth, 6 per population) were collected in April and May of 2004 and homogenized to produce one sample. Soils were analyzed by A & L Western Agricultural Laboratories, 1311 Woodland Ave #1, Modesto, CA 95351.

*Alkaloid Analysis*

Entire root or shoot samples of each individual were ground separately to a fine powder in a coffee grinder and their masses were determined to four decimal places. The ground plant material was wetted with saturated aqueous sodium bicarbonate to ensure that the basic alkaloids would be in the organic soluble free base form. This material was extracted with HPLC grade methanol (200ml) overnight twice at room temperature with mechanical stirring. The solvent of the combined extracts was removed *in vacuo* and the residue was re-dissolved in 0.01M sulfuric acid with heating on a steam bath. The acidic aqueous layer was extracted with five portions of chloroform (50ml) to remove the acidic and neutral compounds while leaving behind the aqueous soluble protonated amines. The acidic aqueous layer was then made basic to pH10 with 6M sodium hydroxide. The deprotonated free bases were then extracted from the basic aqueous layer with five portions of chloroform (50ml) and the solvent from the combined chloroform layers was removed *in vacuo*. This residue consisted entirely of a mixture of basic alkaloids and its mass was determined and used to calculate the percent alkaloid in each sample by mass. Each root alkaloid residue was dissolved in deuterochloroform and a 300 MHz proton NMR spectrum was collected (JEOL Eclipse 300). A portion of each root alkaloid residue was dissolved in minimal chloroform and spotted on a silica gel TLC plate which was developed in 100:1 chloroform: methanol with 1 drop of concentrated ammonium hydroxide added per 15ml of solvent system. Developed plates were visualized using short wave UV absorbance, long wave UV fluorescence and an iodoplatinic acid alkaloid specific spray reagent.

The number and R_f_ values of UV absorbing spots in the TLC matched those spots reacting positively to the alkaloid spray test (i.e. reagent changed color).  NMR showed the same number of N-methyl resonances as there were UV absorbing and spray positive spots in the TLC. Finally, although we could not definitively rule out the possible (but very unlikely) presence of non-UV absorbing and non-alkaloid positive material, the NMR did not contain any large peaks consistent with such compounds, so any impurities were minor (percent level or lower).

The residue after extraction (marc) of the highest mass mainland root sample (14.7070 g with an alkaloid content of 3.71%) was carried through the entire extraction procedure a second time in order to prove that the original extraction was exhaustive. This entire crude base fraction (less than 1mg) was spotted on a TLC plate and sprayed with iodoplatinic acid. The spray test was negative, indicating less than 60μg of basic alkaloid present or less than 4ppm alkaloids in the marc.

The vast majority of the alkaloid compounds found in *E. californica* are bases and can easily be isolated, however two benzophenanthrines known to be present (sanguinarine and chelerythrine) are quaternary ammonium salts and as such are not basic and are water soluble at all pH. TLC and NMR data from the extracted aqueous layers of both roots and shoots indicate that only small amounts of benzophenanthrines were present in the roots, although there were significant amounts present in the shoots. Therefore, while island and mainland shoots had significant water soluble alkaloid content, our analyses are clear with regard to basic alkaloidal differences between island and mainland roots, regardless of the sensitivity of the assays to the benzophenanthrines.

*Diet Choice Experiment*

Gophers were captured using methods and live traps for pocket gophers described by Howard [9]. Two trapping sites were located in coastal Santa Barbara County: the Del Sol Vernal Pool Reserve (721 Camino Corto, Isla Vista, CA 93117: N34°24.530’ W119°52.682’) and a private residence (10696 Calle Quebrada, Goleta, CA 93117: N34°28.046’ W119°59.275’).

The gophers were housed at the Animal Resource Center (6183 Biological Sciences II, University of California, Santa Barbara, CA 93106-5061). Each cage was equipped with a metal nest box (15.2 x 15.2 x 10.2cm), *Care*FRESH^®^ bedding (International Absorbents Inc.), and two metal food bowls (~10.2cm diameter). Although, in the wild, gophers generally obtain water from plant material, some of the captive gophers learned to use sipper-tube rodent water bottles. In addition to two pellets of Purina Rodent Chow no. 5001, vegetables for the gopher’s normal diet included: “Root” material (carrots, turnips, parsnips, potatoes, radishes) and “Shoot” material (carrot tops, beet tops, arugula, dry alfalfa, bean sprouts, and red apple). The gophers acclimatized well to captivity; seven of ten gained weight during captivity with an average increase of 9.36% of initial weight. Three individuals lost weight (average loss of 6.53% initial weight), but they appeared otherwise healthy, and all individuals were held in captivity for over a year following the experiment.

Diet choice trials directly compared the palatability of roots from climatically similar and divergent island and mainland populations. Six of the trials compared similar climatic conditions (i.e. three trials C. Raton vs. Vandenberg and three trials Field Station vs. C.O. Point), and three trials compared roots from island and mainland sites that had contrasting climates (i.e. two Field Station vs. Vandenberg and one C. Raton vs. C.O. Point). Prior to each trial all roots were trimmed of the top ~2cm and any root branches that tapered to less than ~0.3cm in diameter, this had two purposes: 1) it aided in locating root pieces, 2) it removed the exposed ends of the roots which may have had greater loss of volatile chemicals than the rest of the root. Each trial day proceeded as follows:

Normal Daily Diet: 9:00pm – 12:00pm (15hrs) normal laboratory diet

Food Restriction: 12:00pm – 5:00pm (5hrs) all food removed from the cages

Trial Period: 5:00pm – 9:00pm (4hrs) trial root material offered

*Tolerance Experiment Design*

In each population, three transects were established within an area of approximately 400m^2^. At least 16 individuals were located 0.5 – 3m apart along each transect to total ≥48 individuals per population. In anticipation of some sample loss due to gopher damage, mainland populations included 4 extra plants per block (to total 60 individuals). Thus, island populations had 8 control and 8 treatment plants per block (48 per population), whereas mainland populations had 10 control and treatment plants per block (60 per population).

The root damage treatment consisted of a 7.5cm diameter bore at a ~35° angle to a depth of 11 – 14cm (Dutch Mud Soil Auger; Forestry Suppliers, Inc., Jackson, Mississippi, 39201, Stock #76970). This treatment was designed to estimate the geometry of lateral tunnels (short branches to expel soil from feeding tunnels). Mean tunnel diameters for *Thomomys bottae* have been estimated from 6 – 6.5cm [1,2], but we have commonly encountered feeding tunnels at our study sites with diameters of 10cm. Lateral tunnels have been found to be invariant to hillslope angle at a mean angle of ascent of 37° (SE = 3.72° [10]).

The tolerance treatment is demonstrated in Figure S1a and S1b. The plant shown here is telegraph weed (*Heterotheca grandiflora*; not included in study). The “Dutch Auger” used in the experiment is pictured. Figure S2 provides a comparison of the plugged laterals of an actual gopher tunnel system (to the right and below the *H. grandiflora* in Figure S2a) and the plugged hole resulting from the treatment depicted in Figure S1 (to the right of the *H. grandiflora* in Figure S2b). The wilting of the plant in the left photo is a typical reaction to gopher damage that would also occur within an hour of root damage with the “Dutch Auger).

Artificial herbivory treatments have been criticized as insufficiently similar to natural herbivory [11,12], however, the simulated root herbivory treatments discussed in these reviews were intended to mimic small-scale larval insect root herbivory that often involves tunneling in the root cortex or vascular system. The mode and scale of gopher root herbivory makes it more amenable to simulation with a soil auger. Some secondary effects of gopher tunneling may be absent from this treatment (e.g. type of cellular damage, potential nutrient addition, fungal or bacterial inoculation), however, the volume of root material removed from each plant is a good estimate of gopher herbivory. In addition, the treatment applied here compares tolerance to the same root damage treatment in several populations, as opposed to using it as an estimate of plant responses to herbivory (as reviewed in [11,12]).

**References**

1. Miller MA (1957) Burrows of the Sacramento Valley pocket gopher in flood-irrigated alfalfa fields. Hilgardia 26: 431- 452.

**2. Vleck D (1981) Burrow structure and foraging costs in the fossorial rodent *Thomomys bottae*. Oecologia 49: 391- 396.**

**3. Gabet EJ, Reichman OJ, Seabloom EW (2003) The effects of bioturbation on soil processes and sediment transport. Annual Review of Earth & Planetary Sciences 31: 249- 273.**

**4. Hogue CL (1993) Insects of the Los Angeles Basin. Los Angeles, California: Natural History Museum of Los Angeles County.**

**5. Tanowitz BD (1980) Evolutionary patterns in *Hemizonia* DC. section Madiomeris Nutt. (Asteraceae: Madiinae). [PhD Dissertation]. Santa Barbara, California: University of California, Santa Barbara.**

**6. Hunt J (1992) Feeding ecology of valley pocket gophers (*Thomomys bottae sanctidiegi*) on a California coastal grassland. American Midland Naturalist 127: 41-51.**

**7. Miller MA (1948) Seasonal trends in burrowing of pocket gophers (*Thomomys*). Journal of Mammalogy 29: 38- 44.**

**8. Andersen DC (1987) Belowground herbivory in natural communities: a review emphasizing fossorial animals. The Quarterly Review of Biology 62: 261- 285.**

**9. Howard WE (1952) Howard-live trap: A live trap for pocket gophers. Journal of Mammalogy 33: 61-65.**

**10. Seabloom EW, Reichman OJ, Gabet EJ (2000) The effect of hillslope angle on pocket gopher (*Thomomys bottae*) burrow geometry. Oecologia 125: 26- 34.**

**11. Baldwin IT (1990) Herbivory simulations in ecological research. Trends in Ecology and Evolution 5: 91- 93.**

**12. Blossey B, Hunt-Joshi T (2003) Belowground herbivory by insects: Influence on plants and aboveground herbivores. Annual Review of Entomology 48: 521- 547.**
