# Supplementary figures and images for: The Roots of Defense: Plant Resistance and Tolerance to Belowground Herbivory
Source: PLoS One. 2011 Apr 6;6(4):e18463. doi: 10.1371/journal.pone.0018463 (PMC3071833; doi:10.1371/journal.pone.0018463)

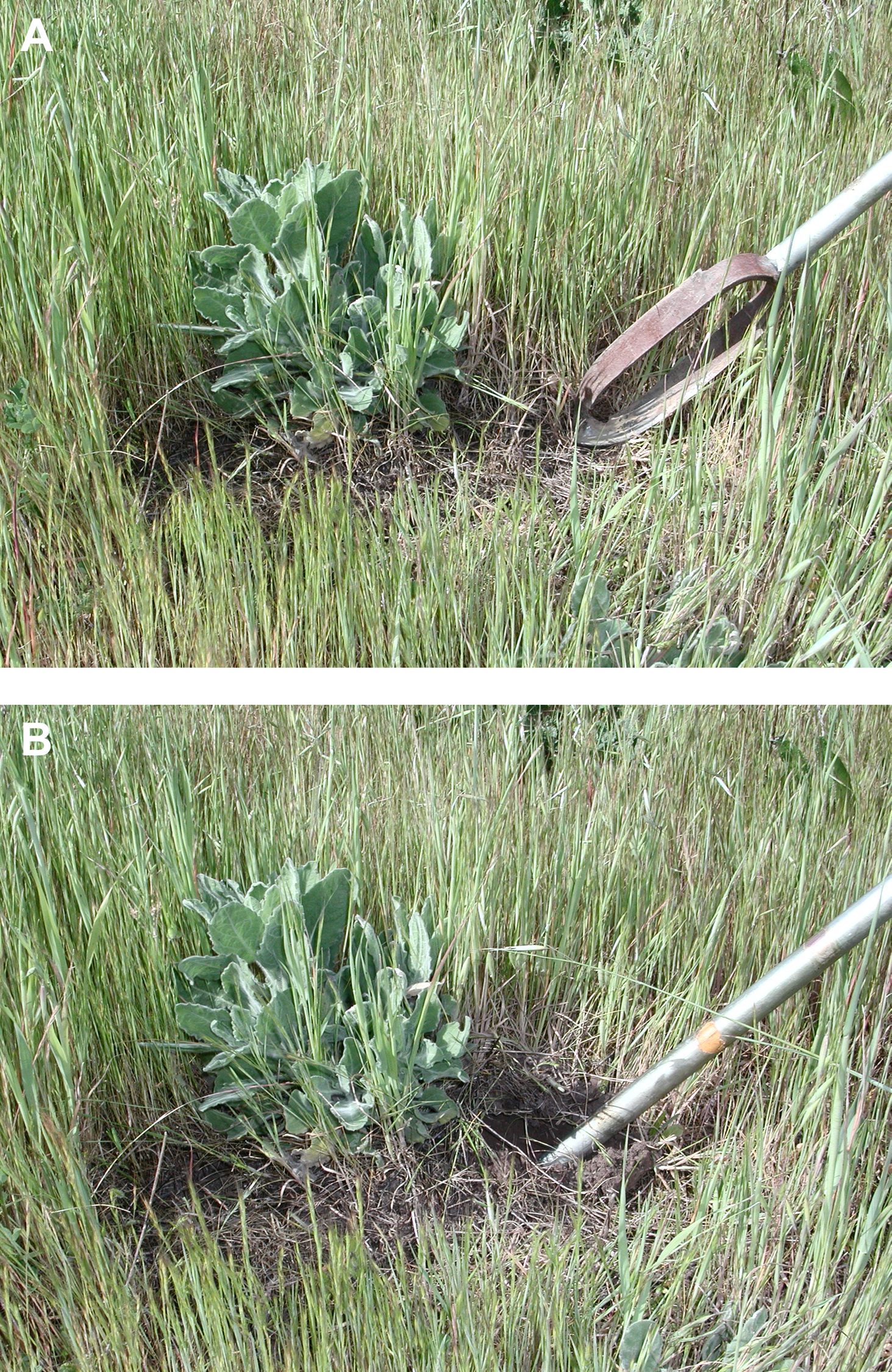

Supplement: Figure S1 — Demonstration of the tolerance treatment. A “Dutch Auger” used in the experiment. B after root damage treatment applied. The plant shown receiving the treatment is telegraph weed (Heterotheca grandiflora, Asteraceae). (TIF) [file pone.0018463.s002.tif]

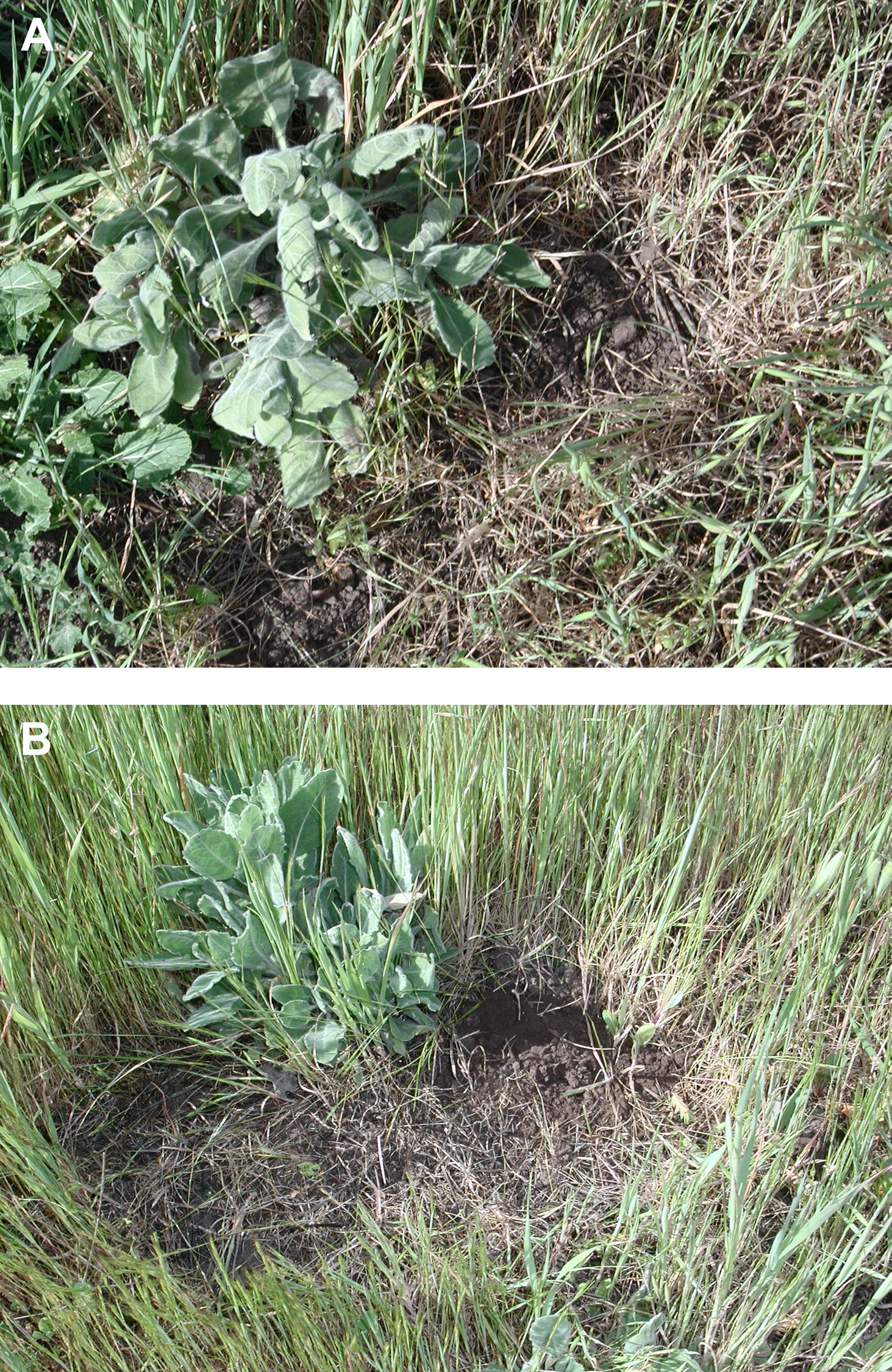

Supplement: Figure S2 — Comparison of actual and simulated root herbivory. A Plugged laterals of an actual gopher burrow (to the right and below plant). B Plugged hole resulting from the treatment depicted in Figure S1 (to the right of plant). The wilting of the plant in Figure S2A is a typical reaction to gopher damage that would also occur within an hour of the treatment in Figure S2B. (TIF) [file pone.0018463.s003.tif]
