# Supplementary material for: The Roots of Defense: Plant Resistance and Tolerance to Belowground Herbivory
Source: PLoS One. 2011 Apr 6;6(4):e18463. doi: 10.1371/journal.pone.0018463 (PMC3071833; doi:10.1371/journal.pone.0018463)
Supplement: Table S1 — Mean annual temperature (°C) and total annual precipitation (cm) at representative mainland and island sites. (DOC) [file pone.0018463.s004.doc]

**Table S1.** Survivorship of *Deinandra fasciculata* and *Eschscholzia californica* in tolerance experiment by site (Note: table does not include mainland pairs dropped from analyses due to gopher mortality).

| ***Deinandra fasciculata*** |  |  |  |  |  |  |  |
| --- | --- | --- | --- | --- | --- | --- | --- |
| **Mainland (Gaviota)** | C **^a^** | T **^a^** | Tot **^a^** | **SC Island (Christy)** | C | T | Tot |
| Initial Census | 26 | 26 | 52 | Initial Census | 24 | 24 | 48 |
| Final Census | 22 | 20 | 42 | Final Census | 24 | 4 | 28 |
| Proportion of Initial | 0.85 | 0.77 | 0.81 | Proportion of Initial | 1.00 | 0.17 | 0.58 |
|  |  |  |  |  |  |  |  |
| **Mainland (Refugio) ^b^** | C | T | Tot | **SC Island (UC Field Stn)** | C | T | Tot |
| Initial Census | 29 | 29 | 58 | Initial Census | 24 | 24 | 48 |
| Final Census | 8 | 9 | 17 | Final Census | 23 | 8 | 31 |
| Proportion of Initial | 0.28 | 0.31 | 0.29 | Proportion of Initial | 0.96 | 0.33 | 0.65 |
|  |  |  |  |  |  |  |  |
| ***Eschscholzia californica*** |  |  |  |  |  |  |  |
| **Mainland (Vandenberg)** | C | T | Tot | **SC Island (C. Raton)** | C | T | Tot |
| Initial Census | 27 | 27 | 54 | Initial Census | 24 | 24 | 48 |
| Final Census | 26 | 26 | 52 | Final Census | 24 | 23 | 47 |
| Proportion of Initial | 0.96 | 0.96 | 0.96 | Proportion of Initial | 1.00 | 0.96 | 0.98 |
|  |  |  |  |  |  |  |  |
| **Mainland (C.O. Point)** | C | T | Tot | **SC Island (UC Field Stn)** | C | T | Tot |
| Initial Census | 24 | 24 | 48 | Initial Census | 24 | 24 | 48 |
| Final Census | 21 | 22 | 43 | Final Census | 24 | 20 | 44 |
| Proportion of Initial | 0.88 | 0.92 | 0.90 | Proportion of Initial | 1.00 | 0.83 | 0.92 |
|  |  |  |  |  |  |  |  |

**^a^** C = Control; T = Root-damaged treatment, Tot = Total individuals, control and treatment

**^b^** High mortality in both control and root damaged plants reflects relatively early senescence at this site. All but one treatment plant survived through the mid-season census.
