# Supplementary material for: The Roots of Defense: Plant Resistance and Tolerance to Belowground Herbivory
Source: PLoS One. 2011 Apr 6;6(4):e18463. doi: 10.1371/journal.pone.0018463 (PMC3071833; doi:10.1371/journal.pone.0018463)
Supplement: Table S2 — Soil data for each mainland and island site (SE Anchorage is on Santa Rosa Island). (DOC) [file pone.0018463.s005.doc]

**Table S2.** 2001 Common Garden Results: Mann-Whitney tests on ranked total number of flowers and fruits (TFF) in undamaged island (I1 – I5) and mainland (M1 – M3) *Deinandra fasciculata* in 2001 (censuses 3 & 4)**^a^**.

| ***Census 3* ^b^** | ***P* = 0.05** |  |  | ***Census 4* ^c^** | ***P* = 0.10** |  |
| --- | --- | --- | --- | --- | --- | --- |
| **Plants** | **TFF** | **Rank** |  | **Plants** | **TFF** | **Rank** |
| I1 | 1029 | 1 |  | I1 | 1142 | 1 |
| I2 | 1012 | 2 |  | I2 | 865 | 2 |
| I3 | 487 | 3 |  | I4 | 381 | 3 |
| I4 | 247 | 4 |  | I3 | 353 | 4 |
| M2 | 109 | 5 |  | M1 | 335 | 5 |
| I5 | 12 | 6 |  | M2 | 271 | 6 |
| M1 | 171 | 7 |  | I5 | 91 | 7 |
| M3 | 0 | 8 |  | M3 | 25 | 8 |

**^a^** **H_0_** = Island TFF </= Mainland TFF; **H_a_** = Island TFF > Mainland TFF

**^b^** **Test for census 3:** *U* = *n*_1_*n*_2_ + *n*_1_(*n*_1_+1)/2 – *R*_1_, where *n*_1_ = Island sample size, *n*_2_ = Mainland sample size, *R*_1_ = 16, *R*_2_ = 20.

*U* = 5(3) + 5(6)/2 – 16 **= 14**; ***U*_0.05(1)3,5_ = 14;** *U*_0.10(1)3,5_ = 13

**^c^** **Test for census 4:** *U* = *n*_1_*n*_2_ + *n*_1_(*n*_1_+1)/2 – *R*_1_, where *n*_1_ = Island sample size, *n*_2_ = Mainland sample size, *R*_1_ = 17, *R*_2_ = 19.

*U* = 5(3) + 5(6)/2 – 17 **= 13**; *U*_0.05(1)3,5_ = 14; ***U*_0.10(1)3,5_ = 13**
