# Supplementary material for: The Roots of Defense: Plant Resistance and Tolerance to Belowground Herbivory
Source: PLoS One. 2011 Apr 6;6(4):e18463. doi: 10.1371/journal.pone.0018463 (PMC3071833; doi:10.1371/journal.pone.0018463)
Supplement: Table S3 — Survivorship of Deinandra fasciculata and Eschscholzia californica in tolerance experiment by site (Note: table does not include mainland pairs dropped from analyses due to gopher mortality). (DOC) [file pone.0018463.s006.doc]

**Table S3.** Mean annual temperature (°C) and total annual precipitation (cm) at representative mainland and island sites.

|  | **Mainland**  **Lompoc** | **Santa Cruz Island**  **Christy Airstrip** | **Mainland**  **S. Barbara Airport** | **Santa Cruz Island**  **Field Station** |
| --- | --- | --- | --- | --- |
| **Year** | **Temp (°C)** | **Temp (°C)** | **Temp (°C)** | **Temp (°C)** |
| 1998-99 | 14.20 | 12.83 | 15.74 | 14.52 |
| 1999-00 | 15.23 | 13.60 | 16.61 | 15.61 |
| 2000-01 | 14.61 | 13.31 | 16.00 | 14.78 |
| 2001-02 | 14.46 | 13.96 | 15.87 | 14.83 |
| 2002-03 | 15.16 | 13.50 | 16.57 | 15.63 |
| **Year** | **Precip (cm)** | **Precip (cm)** | **Precip (cm)** | **Precip (cm)** |
| 1998-99 | 37.59 | 25.58 | 27.97 | 24.41 |
| 1999-00 | 40.06 | 22.56 | 54.23 | 35.26 |
| 2000-01 | 50.75 | 43.97 | 61.70 | 54.91 |
| 2001-02 | 22.56 | 14.96 | 23.34 | 26.04 |
| 2002-03 | 33.71 | 26.87 | 62.05 | 50.27 |
