# Supplementary material for: The Roots of Defense: Plant Resistance and Tolerance to Belowground Herbivory
Source: PLoS One. 2011 Apr 6;6(4):e18463. doi: 10.1371/journal.pone.0018463 (PMC3071833; doi:10.1371/journal.pone.0018463)
Supplement: Table S4 — 2001 Common Garden Results: Mann-Whitney tests on ranked total number of flowers and fruits (TFF) in undamaged island (I1–I5) and mainland (M1–M3) Deinandra fasciculata in 2001 (censuses 3 & 4). (DOC) [file pone.0018463.s007.doc]

**Table S4.** Soil data for each mainland and island site (SE Anchorage is on Santa Rosa Island).

|  | **MAINLAND** |  |  |  |
| --- | --- | --- | --- | --- |
| **Variable** | **Vandenberg** | **Gaviota** | **Refugio** | **C.O. Point** |
| NO_3_ | 11ppm | 4ppm | 5ppm | 6ppm |
| Sand | 87% | 59% | 77% | 65% |
| Silt | 6% | 20% | 10% | 22% |
| Clay | 6% | 20% | 12% | 12% |
|  |  |  |  |  |
|  | **ISLAND** |  |  |  |
| **Variable** | **SE Anchorage** | **Christy** | **C. Raton** | **UC Field Stn** |
| NO_3_ | 6ppm | 7ppm | 5ppm | 5ppm |
| Sand | 63% | 29% | 61% | 39% |
| Silt | 28% | 36% | 24% | 32% |
| Clay | 8% | 34% | 14% | 28% |
